# The Targeting of RNA Polymerase I Transcription Using CX-5461 in Combination with Radiation Enhances Tumour Cell Killing Effects in Human Solid Cancers

**DOI:** 10.3390/cancers11101429

**Published:** 2019-09-25

**Authors:** Mohammed Ismael, Roger Webb, Mazhar Ajaz, Karen J. Kirkby, Helen M. Coley

**Affiliations:** 1 Ion Beam Centre, Advanced Technology Institute, University of Surrey, Guildford, Surrey GU2 7XH, UK; mismael258@gmail.com (M.I.); r.webb@surrey.ac.uk (R.W.); karen.kirkby@manchester.ac.uk (K.J.K.); 2 Faculty of Health and Medical Sciences, University of Surrey, Guildford, Surrey GU2 7XH, UK; m.ajaz@nhs.net; 3 Division of Cancer Sciences, Faculty of Biology, Medicine and Health, The University of Manchester, Manchester M13 9PL, UK

**Keywords:** CX-5461, RNA polymerase I targeting, combination studies

## Abstract

An increased rate of cellular proliferation is a hallmark of cancer and may be accompanied by an increase in ribosome biogenesis and dysregulation in rRNA synthesis. In this regard, CX-5461 has been developed as a novel RNA polymerase I inhibitor and is currently in Phase I/II clinical trials for solid and hematological malignancies. In the present study, interactions between CX-5461 and single-dose X-ray exposure were assessed using isobologram analysis using MTS assay and drug-induced cell death was assessed using flow cytometric, confocal microscopy and Western blot analysis. Combination treatments involving CX-5461 and single-dose X-ray exposure highlighted increased effectiveness compared to individual treatment alone in the CaSki cervical cancer line, with marked synergistic interaction occurring within the low-drug (50 nM) and low-dose radiation range (2–6 Gy). Cell lines challenged with CX-5461 demonstrated the presence of DNA damage, induction of apoptosis, autophagy and senescence alongside high percentages of G2/M cell cycle arrest. In addition, we report preferential sensitivity of ovarian cancer cells with BRCA2 mutation to this novel agent. Taken together, CX-5461 displayed a broad spectrum of activity in a panel of solid cancer cell lines with IC_50_ values ranging from 35 nM to >1 µM. The work described herein identifies the synergistic effects of CX-5461 in combination with X-rays in solid cancers and may also aid in the design of clinical trials involving this novel agent.

## 1. Introduction

The nucleolus is a prominent subcellular organelle involved in a wide range of cellular processes [1,2]. The primary function of the nucleolus is to transcribe and process the 45s pre rRNA transcripts which are located along tandem repeats within nucleolar organizer regions (NORs) located within the short arms of acrocentric chromosomes. The 45s pre rRNA transcripts are processed into the small and large ribosomal subunits within the nucleolus, which are then exported into the cytoplasm and assembled with RNAs and riboproteins to form fully assembled ribosomes. 

RNA polymerase I is exclusively dedicated to the transcription of 45s rRNA genes and has recently become the centre of attention for a number of selective agents aimed at inhibiting the transcription of rRNA for cancer intervention [3,4]. Increased levels of rRNA synthesis has been previously associated with adverse prognosis in cancer [5] and the increase in the number of nucleoli, as well as their size within individual nuclei, has been established as a marker for tumour aggressiveness [6]. 

Several approved anticancer agents have been shown to inhibit rRNA synthesis but these drugs mechanistically do not target the RNA Polymerase I machinery such as cisplatin, 5-FU and actinomycin D (ActD), which at low doses has been shown to inhibit rRNA synthesis through intercalation within guanine and cytosine bases (GC-rich regions) within NORs [7]. In this regard, CX-5461 is a potent and selective anti-cancer agent which has been shown to inhibit rRNA transcription through selective targeting of RNA polymerase I transcription machinery [8]. The mechanism of action of CX-5461 involves the prevention of selectivity factor 1 (SL-1) from interacting with the pre-initiation complex (PIC) and the rDNA promoter site [8]. Previous reports involving CX-5461 showed that in hematological malignancies, p53-dependent apoptosis is induced and also, potent anti-tumour effects were observed in xenograft models [9]. In solid cancers, data from experiments with CX-5461 have shown the induction of autophagy and senescence [8].

For the treatment and management of cancer, the standard regiment of treatment involves surgery, radiation and/or chemotherapy. There are many tumours for which the standard treatment has not changed significantly over the last few years—such as first-line platinum for management of ovarian and cervical cancers. As both these tumour types are associated with poor prognosis in the metastatic phase, we have considered these as part of our study. Moreover, CX-5461 is currently in clinical trial in BRCA mutant breast cancer (Clinical trial identification: NCT02719977) and so we have considered this using an ovarian cancer model with BRCA2 mutation. Although experiments have shown synergistic effects when combining CX-5461 alongside doxorubicin in osteosarcoma cell lines [10], no evidence in the literature exists which explores the effects of combining RNA Pol I inhibitors alongside radiation therapy for the treatment of solid cancers. In the present study, we investigated the effects of combining CX-5461 treatment alongside X-ray radiation with a focus on cervical cancer and have then attempted to identify any beneficial effects of this approach to improve the therapeutic efficacy of targeting Pol I transcription. In addition, we have also considered glioblastoma in our current investigation as this is a tumour type treated with radiotherapy but is associated with a poor prognosis, making it an ideal candidate for new therapeutic options.

## 2. Results

### 2.1. A Panel of Solid Tumour Cell Lines Showed a Spectrum of Sensitivity Towards CX-5461

**Figure 1:**
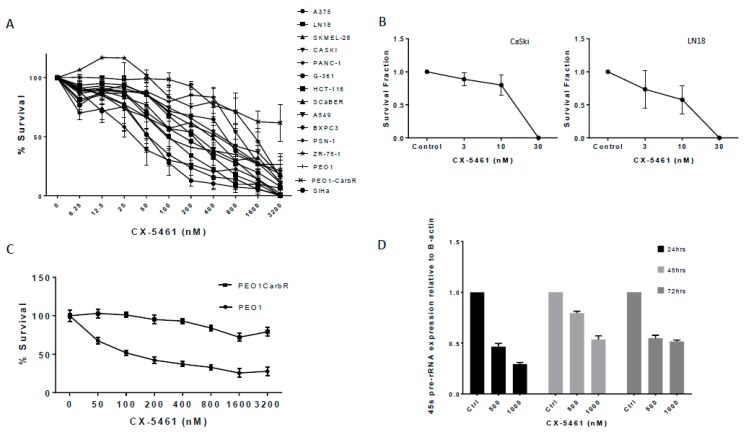
CX-5461 is a potent inhibitor of RNA Polymerase I in solid cancer cell lines. A panel of fifteen solid cancer cells lines was exposed to CX-5461 over 96 h with cell viability assessment using the MTS assay (**A**). Each cell line was set up in triplicate on the day of experiments and repeated on three separate occasions. (**B**) CX-5461 as an anti-proliferative agent. Graphs showing survival fraction values of CaSki (top) and LN18 (bottom) following exposure to CX-5461. Doses of 30 nM were sufficient to prevent the formation of colonies in both cell lines. (**C**) Cell viability data obtained using the MTS assay in PEO1 (BRCA mutant) and PEO1 carboplatin resistant (BRCA wild-type) cell lines showing the difference in sensitivity towards CX-5461. (**D**) qPCR evaluation of 45s pre rRNA transcription of CaSki cell line following exposure to CX-5461 over 72 h. cT values were normalised against the housekeeping gene B-actin. Transcription of 45s pre rRNA was found to significantly decrease (*p*-value < 0.0001) in a dose-dependent manner.

Using the MTS assay with continuous exposure of CX-5461, a panel of 14 solid tumour cell lines investigated (Figure 1A) gave IC_50_ values within the nanomolar range (Table 1), The sensitivity of these solid cancer cell lines was not subject to p53 status, as shown in Table 1; however, CX-5461 did seem to be more selective towards PEO1 cell line harboring a BRCA2 mutation compared to its PEO1 carboplatin-resistant line with essentially wild-type BRCA2 status; (Figure 1C). The selectivity of CX-5461 towards RNA POL I transcription was confirmed using qPCR in CaSki cell line, where the transcription of 45s pre-rRNA was found to decrease in a dose-dependent manner over the course of 72 h (*p*-value < 0.0001) (Figure 1D). Levels of housekeeping genes such as GAPDH and beta-actin were shown not to be affected by CX-5461 as they are under transcriptional control of RNA POL II.

Clonogenic assays revealed the potent anti-proliferative effects of CX-5461. Both LN18 and CaSki cell lines exposed to low doses of CX-5461 resulted in marked reduction of colony formation (Figure 1B). Doses of 30 nM were sufficient to prevent complete colony formation compared to untreated controls.

### 2.2. CX-5461 in Combination with X-rays Show Synergistic Interactions

**Figure 2:**
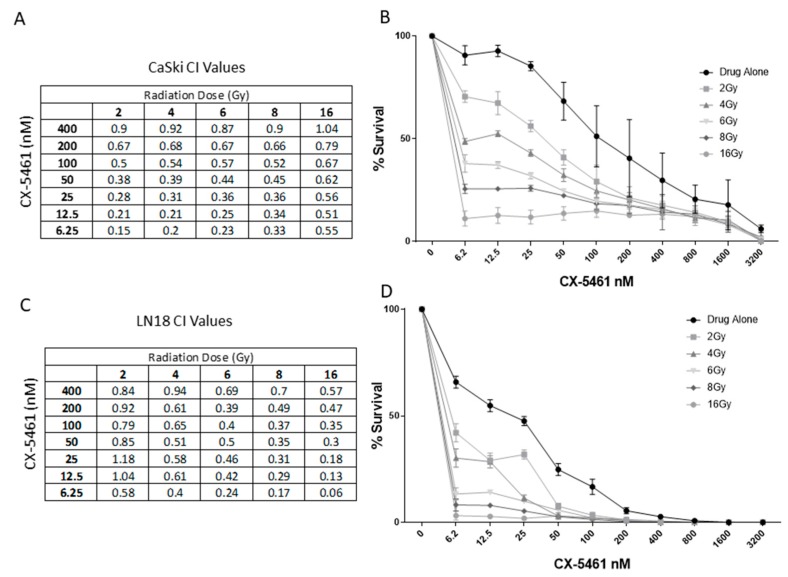
The cytotoxic activity of CX-5461 is enhanced when combined with single-dose X-rays. Combination index values obtained through isobologram analysis for CaSki cell line (**A** and **B**) and LN18 cell line (**C** and **D**) following combination treatments involving CX-5461 and single-dose X-ray exposure (2, 4, 6, 8 and 16 Gy). CI values < 1 were considered synergistic and CI values > 1 were considered antagonistic.

When combined with X-rays, isobologram analysis indicated the most effective combinations of CX-5461 and X-rays were observed in CaSki cell line within the low-drug (6.25–50 nM) and low-radiation dose range (2–6 Gy) (Figure 2A,B), as indicated by the Combination Index (CI) values. LN18 cells also showed effective synergistic interactions between X-ray radiation and CX-5461 treatment but in the low-drug/high-radiation dose range (Figure 2C,D).

### 2.3. CX-5461 Induced Reduced Proliferation, Cell Death and DNA Damage

**Figure 3:**
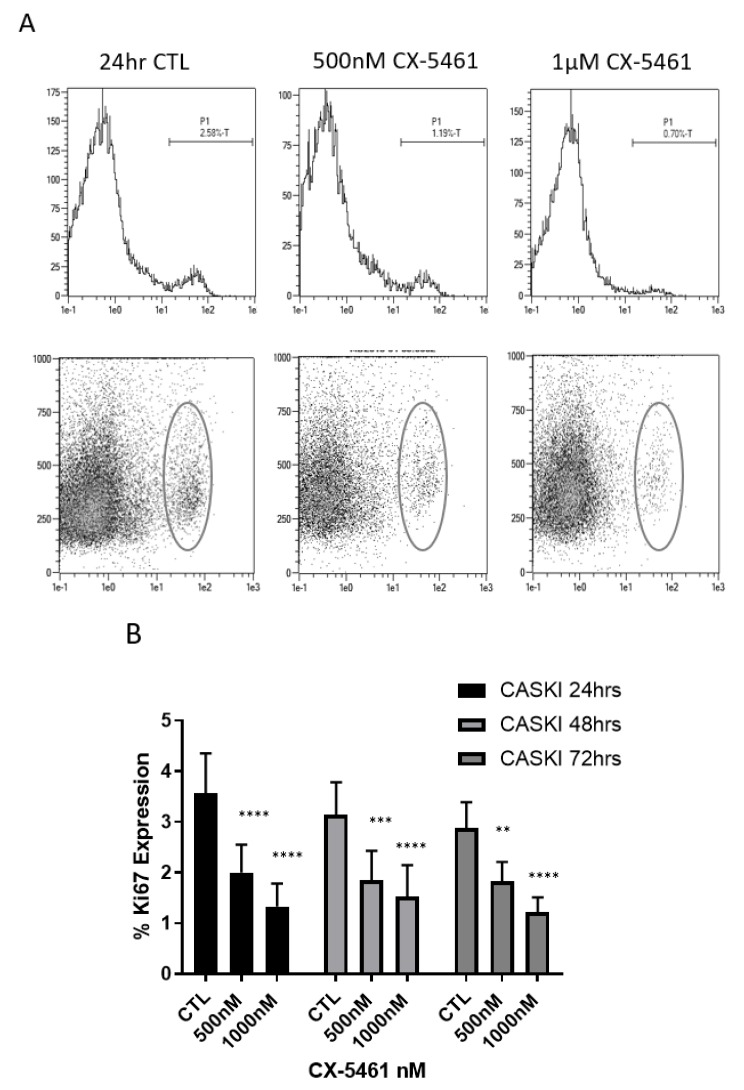
CX-5461 as an anti-proliferative agent. Flow cytometry histogram analysis of CaSki cells probed for Ki67 expression following exposure to sublethal and lethal doses of CX-5461 over 24 h (**A**) revealed that Ki67 expression was seen to decrease in a dose-dependent and time dependent manner (**B**) with a *p*-value of < 0.0001, highlighting the anti-proliferative effects of CX-5461. ** = *p* value < 0.01, *** = *p* value < 0.001 and **** = *p* value < 0.0001.

Ki67 expression, as measured by flow cytometry, was carried in the CaSki cell line over the course of 72 h following low- and high-dose CX-5461 treatment. At each time point, doses of 1 μM significantly reduced the expression of Ki67 in CaSki cells (*p*-value < 0.0001) (Figure 3), indicative of marked anti-proliferative effects.

**Figure 4:**
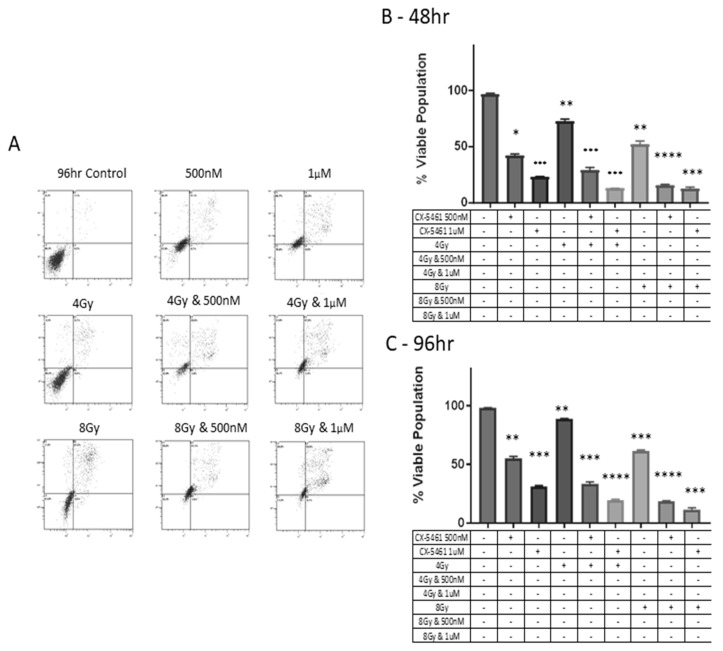
Flow cytometry analysis of combination treatments involving CX-5461 and single-dose X-ray exposure and the induction of apoptosis. Cell death was monitored using Annexin/PI assay at over 96 h. Histogram plots indicate that CX-5461 treatment alone significantly reduced viable cell populations below 50% at doses of 500 nM and 1 µM in CaSki cell line. Exposure to CX-5461 was shown to induce late apoptosis as determined by high-level annexin and high-level PI staining (**A**). When combined with X-rays, the cytotoxic effects of CX-5461 were enhanced when combined with single doses of 4 Gy and 1 µM (*p*-value < 0.001) and further enhanced when using 8 Gy and 1 µM CX-5461 (*p*-value < 0.001). The enhanced effect of combining CX-5461 alongside X-rays was seen as early as 48 h post treatment (**B**) through to 96 h (**C**). ** = *p* value < 0.01, *** = *p* value < 0.001 and **** = *p* value < 0.0001.

Mid-end stage apoptosis was observed in CX-5461-alone-treated CaSki cells (Figure 4A) via annexin V-FITC/PI staining using flow cytometry over 48 h and 96 h. Drug-treated cells significantly reduced cell survival compared to untreated control samples (*p*-value < 0.001). When CX-5461 was combined with 4 Gy and 8 Gy X-rays, cell survival was further decreased compared to either treatment alone (*p*-value < 0.0001) (Figure 4A) and this effect was seen to be consistent over 96 h (Figure 4C). Figure 4B,C show the % viable cell population, i.e., annexin and PI-negative cells.

**Figure 5:**
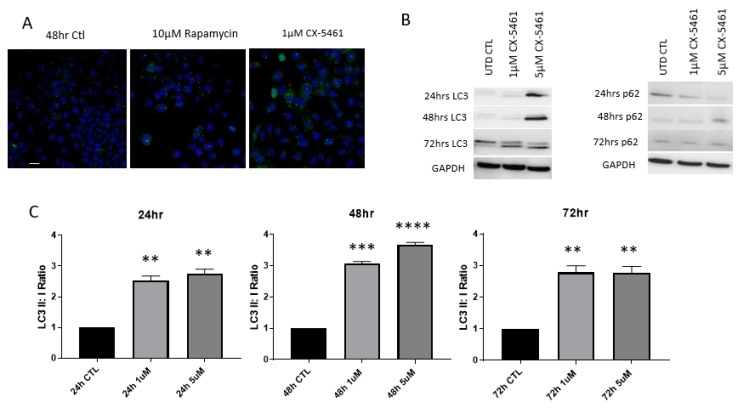
CX-5461 treatment and the induction of autophagy. CaSki cells were treated with 1 µM CX-5461 for 48 h and stained for LC3 puncta using anti-rabbit LC3 (1:1000) and counterstained with DAPI. Rapamycin treatment, a known inducer of autophagy was used as a positive control (**A**). Distinct visualization of autophagolysosomes puncta could be seen in cells exposed to CX-5461, as well as rapamycin. Comparison of the rations of LC3I:LC3II showed significant increases in LC3II:LC3I ratios, indicating the induction of autophagy (**C**). To determine if complete autophagic flux is occurring, LC3 expression was monitored via Western blot analysis, alongside p62 expression over 72 h (**B**). Western data showed complete conversion of LC3I to LC3II accompanied by a reduction in p62 expression over the course of 72 h (**B**). GAPDH was used as loading control to determine relative protein expression. ** = p value < 0.01, *** = p value < 0.001 and **** = p value < 0.0001. Scale bar: 100 μm.

The induction of autophagy was observed in CaSki cells treated with CX-5461 over 72 h. We carried out preliminary experiments to assess the contribution of caspases to the annexin read-outs we were seeing by using the pan-caspase-inhibitor Z-VAD (50 µM). There was evidence of a population of cells that were PI-positive and we then investigated this further to confirm whether these were autophagic (Supplementary Figure S1). Confocal microscopy showed the formation of autophagolysosomes following drug treatment at 48 h (Figure 5A). Complete conversion of LC3I to LC3II was detected using Western blot analysis, alongside a reduction in p62 expression (Figure 5B). Densitometry analysis of LC3I and LC3II bands showed a significant increase in LC3II:LC3I ratios (*p*-value < 0.01) following CX-5461 treatment over 72 h, as shown in Figure 5C. Detailed information on LC3 western blot band intensity ratios in CaSki cell line can be found in Supplementary Table S1.

**Figure 6:**
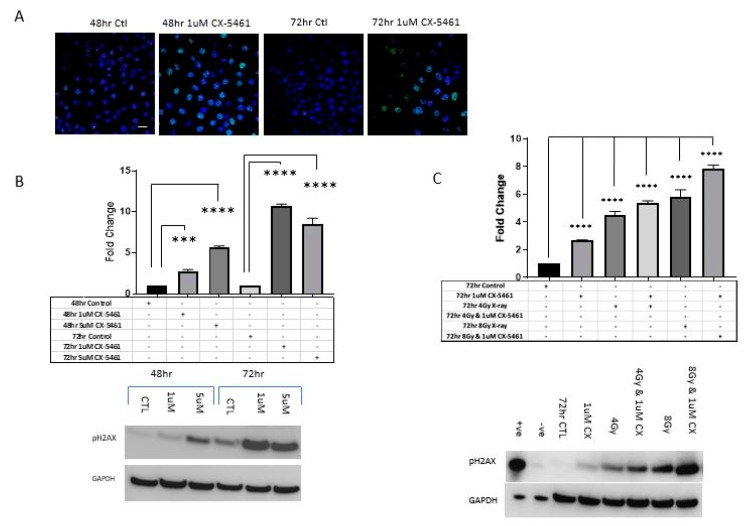
CX-5461 exposure induced DNA damage. CaSki cells were treated with 1 µM CX-5461 over 72 h and visualized using confocal microscopy, DNA damage was marked by γH2AX staining (**A**) (anti-rabbit γH2AX 1:1000 (green) DAPI (blue), distinct green foci could be seen in CX-5461-treated samples, indicating the presence of double-stranded DNA breaks. The expression of γH2AX was found to increase in a dose- and time-dependent manner (*p*-value < 0.0001) (**B**). CaSki cells treated in combination with CX-5461 and single-dose X-rays also resulted in increased expression of γH2AX, as marked by Western blot analysis (**C**). Densitometry analysis showed that exposure alone to CX-5461 or radiation induced γH2AX expression but combination of 4 Gy and 1 µM CX-5461 and 8 Gy and 1 µM CX-5461 significantly increased γH2AX expression compared to either agent alone (*p*-value < 0.0001) (**C**). *** = *p* value < 0.001 and **** = *p* value < 0.0001. Scale bar 100 μm.

CX-5461-induced DNA damage was measured using p-γ-H2AX levels through confocal microscopy alongside Western blot analysis (Figure 6A–C). Both 48 h and 72 h treatment with CX-5461 induced visible γ-H2AX foci formation compared to untreated controls (Figure 6A). Western blot analysis revealed a dose- and time-dependent increase in -γ-H2AX expression over 72 h following CX-5461-alone treatment (Figure 6B), when combined with 4 Gy and 8 Gy X-rays, the induction of -γ-H2AX was stronger compared to either treatment alone (Figure 6C).

**Figure 7:**
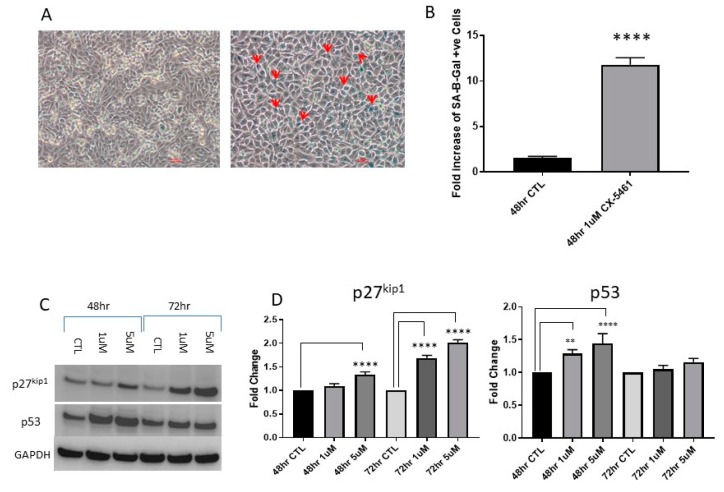
CX-5461 exposure induced cellular senescence. A375 cells where treated with 1 µM CX-5461 and stained for the presence of B-galactosidase following 48 h CX-461 treatment and imaged using brightfield microscopy (**A**). Cells which have entered into a senescent state are marked by red arrows, (magnification 20×). Analysis of blue pixels in treated and untreated samples using ImageJ software showed a significant increase in senescence-associated b-galactosidase-positive cells in CX-5461-treated melanoma cell line (*p*-value < 0.0001) (**B**). Western blot analysis of cell-cycle-associated markers p27^kip1^ and p53 (**C**), p53 induction could be seen as early as 48 h post CX-5461 treatment (*p*-value < 0.0001) in CaSki cell line and strong p27 induction could be observed at 72 h post CX-5461 treatment (**D**). ** = *p* value < 0.01 and **** = *p* value < 0.0001.

Treatment with CX-5461 for 48 h in A375 melanoma cells caused a significant increase in cells staining positive for B-galactosidase (*p*-value < 0.0001), a biomarker indicative of the senescent phase of the cell cycle, compared to the untreated controls (Figure 7A). Analysis of treated and untreated samples showed a >10-fold increase in cells staining positive for SA-B-Gal in A375 cell line (Figure 7B).

### 2.4. Cell Cycle Associated Effects Following CX-5461 Exposure

A marked induction of p53 was observed in wild-type p53 CaSki cells following low- and high-dose CX-5461 exposure at 48 h (*p*-value < 0.001) and also at 72 h (*p*-value < 0.0001) (Figure 7C,D). p27^kip1^ is a marker used to assess the progression of cells through the G1/S transition phase of the cell cycle, induction of p27^kip1^ was observed in CaSki cell line following CX-5461 exposure over 72 h (Figure 7C).

**Figure 8:**
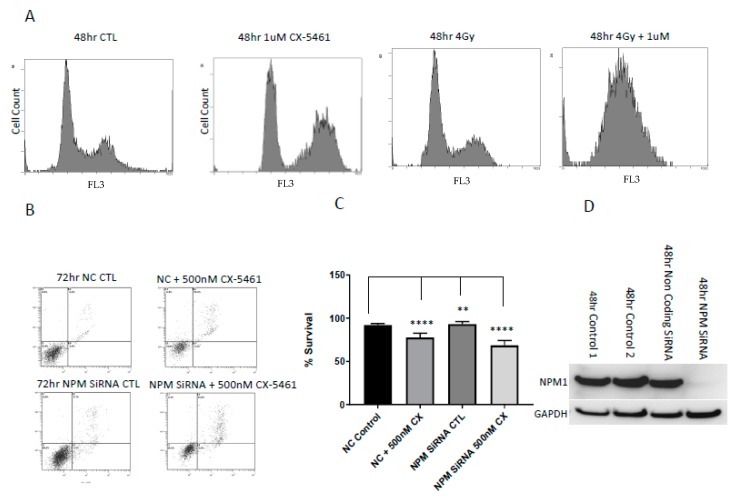
Cell cycle analysis of CaSki cells exposed to CX-5461 alone, radiation alone and in combination for 48 h showed that treatment with CX-5461 alone for 48 h induced strong G2/M arrest compared to radiation-alone treatment, combined treatment resulted in the abolition of the cell cycle, as shown by propidium iodide staining using flow cytometry (**A**). (48 h CaSki control cell cycle values: G0-6%, G1-51%, S-15% G2M-28%. The 1 µM CX-5461 cell cycle values: G0-3%, G1-41%, S-6%, G2M-49%. 4 Gy-radiation-alone cell cycle values: G0-0.8%, G1-53.2%, S-9.2%, G2M-29.4%. Combined 1 µM CX-5461 and 4 Gy X-rays resulted in complete abolition of G2M blockade). *NPM1* silenced cells are more sensitive to RNA Pol I inhibition. (**B**,**C**) Flow cytometry (annexin assay) showed that NPM1 silenced cells were found to be more sensitive towards CX-5461 treatment compared to non-coding treated CaSki cells (*p*-value < 0.0001). CaSki cells were transiently silenced for nucleophosmin (NPM1). Knock down was confirmed through Western blot analysis (**D**). ** = *p* value < 0.01 and **** = *p* value < 0.0001.

Treatment of CaSki and A375 cells with sub-lethal and lethal doses of CX-5461 induced a marked G2/M cell cycle arrest, as shown through propidium iodide staining using flow cytometry. The percentage of cells in G2/M arrest increased from 28% in untreated controls to 49% in CaSki cells treated with 1 µM CX-5461 for 48 h (Figure 8A). Similarly, in A375 cells, the percentage of cells in G2/M in untreated samples was 28% and this increased to 51% following 1 µM CX-5461 48 h exposure (Supplementary Figure S2). When CX-5461 was combined with 4 Gy X-rays in CaSki cells over 48 h, cell cycle analysis reported a complete abolition of the G2/M blockade (Figure 8A).

### 2.5. Low Levels of the Nucleolar Protein Nucleophosmin Enhances the Cell Death Response of Cancer Cells to the Effects of CX-5461

Nucleophosmin (NPM1) is a prominent, multifunctional nucleolar protein. We wished to assess the presence of NPM1 on the nucleolar-targeting agent CX-5461 as a possible marker of sensitivity to this agent. NPM1 was transiently silenced using siRNA in the CaSki cell line exposed to CX-5461 for 72 h. Annexin/PI assay showed that NPM1 silenced cells treated with 500 nM CX-5461 were significantly sensitised to the effects of CX-5461 compared to non-coding treated control samples treated with the same dose of CX-5461 (*p*-value < 0.005) (Figure 8B,C). Knockdown of NPM1 was confirmed through Western blot analysis (Figure 8D). The findings of these experiments were, therefore, suggestive of NPM1 offering a protective effect versus CX-5461 whilst reduced/low levels enhance the cytotoxic effects of this agent. 

## 3. Discussion

The data presented herein shows that CX-5461 is a potent anti-tumour agent with broad spectrum effects seen in a panel of human solid tumour cell lines, Table 1. Further, this effect is enhanced in a synergistic manner when treatment is combined with radiation therapy. Previous findings by Drygin et al. [8] and by Hein et al. [11] showed that CX-5461 was most potent against cell lines derived from human hematological malignancies with wild-type p53 status, whereas median IC_50_ levels in those cell lines with mutated p53 were shown to be several-fold higher. No clear relationship could be established between p53 status of the cell lines in our panel and their respective response to CX-5461 exposure. Expression levels of 45s pre-rRNA were shown to decrease following treatment with CX-5461 for A375 cells and for CaSki cells. Drygin et al. [8] also showed CX-5461 induced a reduction in 45s pre-rRNA for A375 cells, in support of the role of this agent in targeting the transcription of genes under the control RNA polymerase I.

The human cervical cancer cell line CaSki, which was a point of focus in the present study, is positive for Human Papilloma virus HPV16. The virus carries two oncogenes which code for the oncoproteins E6 and E7 which bind to and target tumour suppressors p53 and Rb, respectively. In the present study, CX-5461 combined with X-rays gave a synergistic response in all cell lines examined. The most highly synergistic interactions were seen for the CaSki cell line in the low-drug (50 nM) low-radiation dose (2-6 Gy), which differed from the LN18 and A375 cells where optimal synergy was seen for the low-drug: high-radiation dose. Previously, docetaxel has been shown to radio-sensitise CaSki cells [12] and also, platinum agents alongside 5 Gy radiation have been reported to show synergistic effects in SiHa cervical cancer cells [13]. Radio-sensitizing effects in CaSki cells have been shown using the PI3-kinase inhibitor LY294002. Whereas LY294002 alone did not decrease overall cell viability, a pre-treatment at 0.5, 2 or 6 h prior to radiation produced a pronounced radio-sensitisation effect, *p* < 0.0001 [14]. Another study showed that long-term exposure of HeLa cervical cancer cells to cisplatin and E6/E7 SiRNA induced cellular senescence and apoptosis in vitro. In the same study, in vivo effects were also seen with the same combination with induction of apoptosis, senescence and anti-angiogenic effects [15].

Our findings of the G2M blockade induced by CX-5461 treatment are in agreement with the work of others using ovarian cancer [16], glioblastoma [17], acute myeloid leukaemia [9] and osteosarcoma [18] cell line models. The G2M phase of the cell cycle is the most radiosensitive and thus, this appears to underlie the marked synergistic effects seen when cells are treated with CX-5461 and radiation in combination, as we saw an abolition of this cell cycle phase when cells were treated with this approach.

γH2AX has been shown to be a good marker for the presence of DNA double-strand breaks following exposure of cells to ionizing radiation [19]. Our data clearly showed that CX-5461 treatment of human cancer cell line CaSki (and in LN18 and A375) resulted in antiproliferative effects (Ki67 data) and DNA damage via the formation of double-stranded DNA breaks, as shown by the marker γH2AX. These effects were further enhanced via X-rays when used in combination with CX-5461. Interestingly, Quin et al. [20] showed that γH2AX foci formation or induction at the protein level was not seen after short-term (6 h) exposure of BJ-T cancer cells to CX-5461. However, long-term exposure (48 h) resulted in γH2AX foci following treatment with 1 µM CX-5461 [20] and are in agreement with our findings.

The synergy observed in CaSki cells is important since the ideal clinical setting for cancer treatment is to deliver the lowest dose of radiation and a low dose of chemotherapy to avoid toxic side effects, whilst maximising the anti-tumour effect. Given the findings of our study, further investigation that looks at an in vivo model of human cervical cancer is warranted, but is beyond the scope of the current study that set out to consider in vitro effects of the treatment modality.

Since CX-5461 is currently in phase I/II clinical trials in Canada for breast cancer (Clinical trial identification: NCT02719977), treatment combination studies involving BRCA1/2 mutant cell lines could be informative. CX-5461 has previously been shown to target BRCA mutations leading to DNA damage via faulty homologous recombination and DNA-PK non-homologous end-joining for repair. Our experimental approach used PEO1 BRCA2 mutant cells alongside the PEO1CarbR carboplatin-resistant counterpart in the assessment of growth inhibition effects of CX-5461. The PEO1CarbR cells—which possess an acquired secondary BRCA2 mutation conferring full-length BRCA2 (via a single base substitution) [21] showed a greater than 30-fold cross-resistance to CX-5461 relative to the parental PEO1 cells. Clearly, the restoration of BRCA2 to the PEO1CarbR cells is an important observation and emphasizes that CX-5461 preferentially targets BRCA mutant cells. This has been described previously by [22], who showed synthetic lethality towards tumours with BRCA1/2 deficiencies when treated with CX-5461. The study showed that CX-5461 was highly toxic towards BRCA2 knockdown in HCT-116 cells with high levels of apoptosis compared to the BRCA2 wild-type counterpart. Although radiation is rarely used in the management of ovarian cancers, future investigation should focus on a combination of CX-5461 with radiotherapy for this poor prognosis tumour type. 

Other parts of the present study examine cell fate following treatment with CX-5461. There are reports that cancer cells derived from solid tumours enter a senescent-like state following treatment with CX-5461 [8,16,20]. In A375 human malignant melanoma cells, we too showed that SA-β-gal (senescence-associated β-galactosidase) was significantly increased following CX-5461 treatment for 48 h. In regard to programmed cell death (PCD), i.e., PCD types I or II, CX-5461 was shown by Bywater et al. [9] to produce p53-dependent apoptosis in hematological malignancies. In contrast, cancer cell lines derived from solid tumours were reported to display autophagic cell death and senescence in A375, and MIAPaCa-2 cells [8] and also, in osteosarcoma cells [10]. In the present study, autophagic flux, as a measure of autophagic degradation, was seen for SKMEL-28 and CaSki cells but was not apparent for A375 and LN18 cell lines, as seen by incomplete conversion from LC3I to LC3II following treatment with CX-5461. The conversion of non-lipidated LC3I to its lipidated form LC3II represents conjugation with phosphatidylethanolamine (PE) and the amount of LC3-II is closely correlated with the number of autophagosomes [16]. As LC3II is itself degraded during autophagy, then it alone cannot serve as a solid indicator of autophagic flux. P62 (SQSTM1/ sequestosome 1) can bind to LC3 and acts as a selective substrate for autophagy and hence, we assessed this protein as part of our investigation. Regarding apoptosis (caspase-dependent PCDI) we were able to demonstrate a clear dose-dependent increase in CaSki cells following treatment with CX-5461, which was also apparent at highly significant levels following combinations of drug and X-rays. These results are in agreement with Xu et al. [22], who showed that apoptosis could be induced in HCT116 colorectal carcinoma cells and also, in a range of glioma cell lines [23]. The findings are, however, in contrast to the original paper by Drygin et al. [8] who failed to see any signs of apoptotic cell death in their panel of human cancer cell lines derived from solid tumours. However, in that report, the authors looked at cleavage of PARP and caspase activation via Western blot analysis up to 48 h. 

Studies have identified over 250 proteins to be associated with the nucleolus [24]. One such protein is nucleophosmin (B23/NPM1), a multifunctional protein involved in ribosome biogenesis, genomic stability, DNA repair and autophagy [25,26,27]. It has been shown that NPM1 is overexpressed in drug-resistant ovarian cancer cells using an in-depth proteomic mass spectrometry study [28,29]. In addition, in high-grade ovarian serous cancer [30], a poorer patient outcome was associated with a significant overexpression of nuclear NPM1 and in transformed ovarian cancer cells [31], elevated levels of NPM1 were reported. Silencing of NPM1 expression by Kalra et al. [31] led to aberrant double-strand break repair and p53/p21-mediated apoptosis.

A meta-analysis by Chen et al. [30] looked into the prognostic significance of NPM1 in solid tumour types and concluded that high levels correlated with overall poor survival and tumour aggressiveness. In the present study, we showed that knockdown of NPM1 sensitized CaSki cells to the effects of CX-5461. In the proteomic study by Cruz et al. [28], PEO1CarbR cells expressed more NPM1 than the parental PEO1 line, in line with the relative sensitivity to CX-5461, pointing to a protective role of the protein to the effects of this drug. Olausson et al. [31] silenced NMP1 in human glioma cells which, when treated with the RNA polymerase I inhibitor Actinomycin D, sensitized cells to drug-induced apoptosis, in some agreement with our findings. This warrants further investigation of the sensitivity of cell lines with varying levels of NPM1 to the effects of CX-5461+/− radiation but this is beyond the scope of the current paper.

## 4. Methods and Materials

### 4.1. Cell Lines and Reagents

CaSki and LN18 cell lines were obtained from AATC and cultured in RPMI 1640 or Dulbecco’s Modified Eagle’s Media and supplemented with either 10% FBS or 5% FBS and 1% penicillin/streptomycin and 1% l-glutamine. The media used to maintain the cell lines in the panel used are shown in Supplementary Table S2.

CX-5461 was purchased in lyophilized form from Selleck Chemicals (Houston, TX, USA). The powder was dissolved in 50 mM NaH_2_PO_4_ to produce 10 mM CX-5461 stock solution (pH 4.5) and diluted directly into growth media for working concentrations prior to treatment. Rapamycin was purchased from Sigma Aldrich (HaverHill, UK).

### 4.2. Cell Viability Assay, Clonogenic Assay

Cell lines were seeded in 96-well plates at a density of 3 × 10^3^ cells/well and treated with a range of CX-5461 concentrations for 96 h and cell viability was measured using [3-(4,5-dimethylthiazol-2-yl)-5-3(carboxymethoxyphenyl0-2-(4-sulfophenyl)-2H-tetrazolium] (MTS) assay according to the manufacturers protocol. A quantity of 100 μL of Cell Titre 96 Aqueous Non-Radioactive Cell Proliferation (MTS) Assay (Promega, Southampton, UK) was then diluted to 1:10 using RPMI 1640 media and added to each well, including empty wells for use as a background control. Optical density was measured using a Variskan Flash plate reader (Thermo Scientific, Altrincham, UK) at 492 nm wavelength. Each MTS assay was performed in triplicate and repeated at least three times. For clonogenic assays, cells were grown in 6-well plates and following treatment, incubated for up to 14 days. Colonies were fixed in 50% ethanol in PBS and stained with 5% crystal violet in PBS (Sigma Aldrich, HaverHill, UK). Colonies with more than 50 cells were counted and survival fractions were determined taking into consideration the plating efficiency for all treatment modalities based on triplicate experiments. Calculations were based on established protocols.

### 4.3. Flow Cytometry Analysis

The Annexin V-FITC Apoptosis Detection Kit (CBA059-Calbiochem, San Diego, CA, USA) was used to detect cell membrane alterations which accompany cell death using flow cytometry. The assay was carried out using the manufacturer’s guidelines following the conventional Annexin V Binding protocol. Flow cytometric analysis was performed using a Beckman Coulter Epics Flow Cytometer using FL1 and FL3 laser lines.

### 4.4. Western Blot Analysis

Whole cell lysates were prepared from harvested cells using radioimmunoprecipitation (RIPA) buffer (Life Technologies, Warrington, UK) containing Halt phosphatase and protease inhibitors (Thermo Scientific, Altrincham, UK). The total concentration of protein was determined using Peirce BCA Protein Assay Kit (Life Technologies, Warrington, UK) following the manufacturer’s instructions. The following antibodies were used: anti LC3 (Cell Signaling Technology, Beverly, MA, USA), anti-p62 (Cell Signalling Technology), anti-p53 (DO1) (Santa Cruz, Dallas, TX, USA), anti-p27^kip1^ (Santa Cruz, Dallas, TX, USA), anti-pγH2AX (Cell Signalling Technology) and anti-GAPDH (Origene, Cambridge, UK). Densitometry was performed using UVP imaging software to quantify relative amounts of proteins detected on Western blot membranes. Detailed information can be found in Supplementary Figure S3.

### 4.5. Quantitative Polymerase Chain Reaction

RNA was extracted from drug-treated cells using RNeasy Plus Micro kit (Qiagen, NV, Venlo, the Netherlands). cDNA was produced using Cloned AMV reverse transcriptase First-Strand cDNA Synthesis Kit (Fisher Scientific, Loughborough, UK). qPCR analysis was performed using the Stratagene Mx3005p qPCR machine (Agillent Technologies, Cheshire, UK) using SYBR Green fluorescence to quantify the amount of PCR product. The following primers were used in qPCR experiments: human 45s pre rRNA: FWD 5’-CCGCGCTCTACCTTACCTACCT-3’, REV 5’-GCATGGCTTAATCTTTGAGACAAG-3’, human β-actin FWD 5’-CGTCACCAACTGGGACGACA-3’, REV 5’-CTTCTCGCGGTTGGCCTTGG-3’, human glyceraldehyde-3-phosphate dehydrogenase (GAPDH) FWD 5’-CTCCTGTTCGACAGTCAGCC-3’ and REV 5’-TTCCCGTTCTCAGCCTTGAC-3’. Results were expressed relative to β-actin transcripts as in the internal control. 

### 4.6. Transient NPM1 RNA Silencing

CaSki cells plates into 6-well plates at a density if 1 × 10^5^ cells/mL. Following incubation, cell media were removed and cells were washed briefly with PBS and aspirated and replaced with either NPM1 SiRNA (Dharmacon–GE Healthcare, Lafayette, CO, USA) reagent (4861-J-015737-05 NPM1) or non-coding control SiRNA (Dharmacon–GE Healthcare, 6591-J-017386-05 SNAI2) for 4 h, after which, the silencing media was removed and replaced with media containing 20% FBS and left to incubate overnight. The following day, the 20% FBS media was aspirated and replaced with normal cell culture media (RPMI 1640 10% FBS) and incubated for a further 48 h. Silencing of NPM1 was confirmed using Western blots by probing membranes for NPM1 (Santa Cruz, Dallas, TX, USA).

### 4.7. Ki-67 Expression Analysis

Cells were seeded in 6-well plates at a density of 1 × 10^5^–2.5 × 10^5^ cells/mL and allowed to adhere to the surface overnight at 37 °C. Following incubation, cells were treated with a low- and high-dose of CX-5461 (500 nM and1 μM) over 72 h. After exposure, cells were harvested and washed in 1× PBS, centrifuged at 2000 rpm for 2 min. The cell pellet was resuspended and fixed at room temperature for 15 min using Reagent A of the Fix and Perm Kit (GAS004-Life Technologies, Warrington, UK) and neutralized with binding buffer and centrifuged again for 2 min at 2000 rpm. The cell pellet was resuspended in 90μL of Reagent B and incubated with a 1:10 dilution of Ki-67 antibody (130-100-290-Miltenyi Biotec, Woking, UK) at room temperature for 20 min. Buffer was added to each well and again centrifuged for 2 min at 2000 rpm followed by resuspension in PBS for analysis by flow cytometry.

### 4.8. Immunofluorescence Staining of LC3 and γH2AX

Cells were grown in 8-well chamber slides (BD Bioscience, Wokingham, UK) and allowed to adhere overnight. For microtubule-associated protein 1 light chain (LC3) staining, permeabilization and fixation was done using 100% ice-cold methanol for 5 min at −20 °C, followed by three separate washes using 1× PBS 0.1% Tween-20 washes. Cells marked with γH2AX were permeabilised with 4% paraformaldehyde and fixed with 0.1% Triton X-100 for 10 min at room temperature. Cells were blocked to prevent non-specific binding in 1% BSA in 1× PBS containing 0.1% Tween-20 and 10% Goat serum for 30 min at room temperature. Primary antibody was diluted in 1% BSA PBS 0.1% Tween-20 (1:200 dilutions) and added to chamber slides and incubated at 4 °C overnight in humidified chambers. For autophagy experiments, positive controls were treated with 10 μM rapamycin (Sigma-Aldrich, HaverHill, UK). Cells were washed and incubated with goat anti-rabbit Alexa Fluor 488 (Abcam, Cambridge, UK). Secondary antibodies (1:600) were diluted in 1% BSA 1× PBS and incubated for 1 h at room temperature. Cells were then washed and Vectashield Hardset Mounting Media (Vector Laboratories, Peterborough, UK) containing DAPI was added dropwise onto the glass slide and a glass coverslip was placed on top and allowed to cure for 15 min at room temperature. DAPI served as a counterstain to mark the nucleus of cells. The slide was imaged using a Nikon A1M confocal microscope and using the NIS element acquisition software (64 bit) (Nikon, Kingston upon Thames, UK).

## 5. Conclusions

Taken together, we show data that supports the use of CX-5461 as a novel antitumour agent that is capable of inducing, apoptosis, autophagy, DNA damage and senescence in cancer cell lines derived from solid human tumours. Further, our data supports the use of this agent in future clinical trials used in combination with radiation therapy in solid human tumours.

## Figures and Tables

**Table 1:** IC_50_ values of solid cancer cell lines exposed to CX-5461 over 96 h alongside their respective p53 status. Each cell line was exposed to CX-5461 in triplicates on the day of experiments and also on three separate occasions with different passage numbers for each separate repeat.

Cell Line	IC_50_ nM and SD	p53 Status
PANC-1	35 (−/+ 2.20)	Mut
A375	53 (−/+ 2.28)	WT
G-361	55 (−/+ 1.58)	WT
PEO1	98 (−/+ 4.32)	Mut
LN18	104 (−/+ 3.01)	Mut
A549	169 (−/+ 3.74)	WT
HCT-116	178 (−/+ 2.54)	WT
SKMEL-28	188 (−/+ 4.70)	WT
SCaber	215 (−/+ 2.1)	Mut
SiHa	229 (−/+ 5.68)	WT
BXPC3	257 (−/+ 2.65)	Mut
CaSKI	408 (−/+ 4.08)	WT
ZR-75-1	506 (−/+ 1.69)	WT
PSN-1	656 (−/+ 13.49)	Mut
PEO1CarbR	>3200 (−/+ 5.77)	Mut

## Supplementary Materials

The following are available online at https://www.mdpi.com/2072-6694/11/10/1429/s1, Figure S1: Annexin/PI assay involving exposure of A375 cells to CX-5461 in combination with Z-VAD, Figure S2: Cell cycle analysis of A375 cells exposed to CX-5461 and table of cell line culture medium, Figure S3: Detailed information of western blot analysis, Table S1: CaSki LC3 western band intensity ratios, Table S2: List of solid cancer cell lines used for development of panel exposed to CX-5461.
