# Supplementary material for: The Targeting of RNA Polymerase I Transcription Using CX-5461 in Combination with Radiation Enhances Tumour Cell Killing Effects in Human Solid Cancers
Source: Cancers (Basel). 2019 Sep 25;11(10):1429. doi: 10.3390/cancers11101429 (PMC6826960; doi:10.3390/cancers11101429)
Supplement: Supplementary file 1 [file cancers-11-01429-s001.pdf]

Supplementary Materials

The Targeting of RNA Polymerase I Transcription Using CX-5461 in Combination with Radiation Enhances TμMour Cell Killing Effects in HμMan Solid Cancers

Mohammed Ismael, Roger Webb, Mazhar Ajaz, Karen J. Kirkby and Helen M. Coley

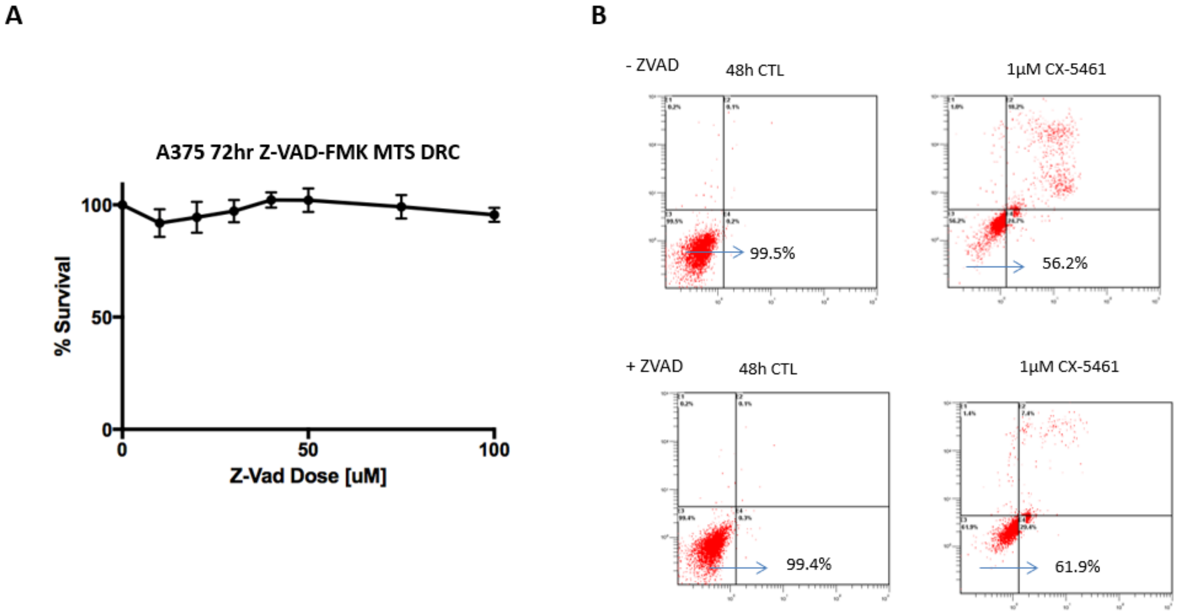

Figure S1. Annexin/PI assay involving exposure of A375 cells to CX-5461 in combination with Z-VAD. (**A**) MTS dose response assay of A375 cells treated with increasing concentrations of Z-VAD-FMK caspase inhibitor to assess non-toxic tolerable doses. A dose of 50 μM Z-VAD was selected for use. (**B**) nnexin V-FITC/PI assay of A375 cells treated with CX-5461 for 48 h with or without Z-VAD-FMK; the percentage viable population of cells (i.e., PI and Annexin negative) are indicated in the bottom left hand quadrant of each panel cytogram. In Z-VAD untreated A375 cells challenged with a lethal dose of 1 μM CX-5461, an apoptotic population (at mid-late phase) is indicated in the top right-hand quadrant. The bottom left hand quadrant also migrates to a more PI positive population. In the presence of Z-VAD treatment with the same dose of CX-5461 the apoptotic (caspase-dependent) population is not present, as expected. However, the PI migration of the control population remains the same indicating that any changes present are not due to a caspase-dependent cell death population and are more indicative of non-caspase dependent programmed cell death.

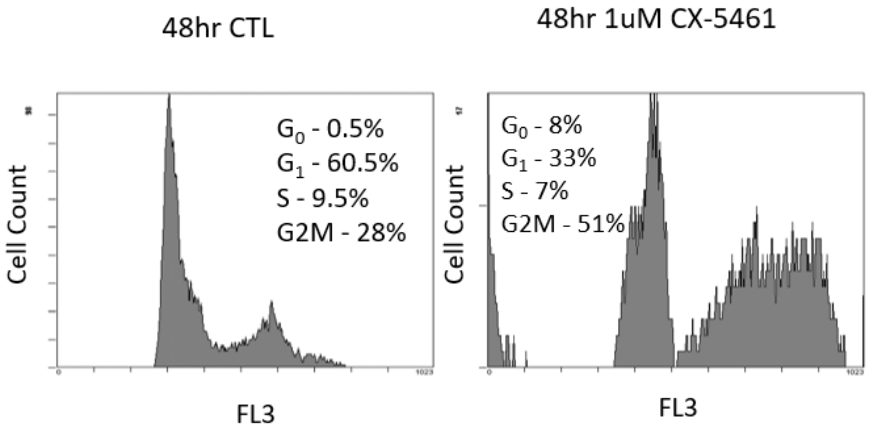

Figure S2. Cell cycle analysis of A375 cells exposed to CX-5461 and table of cell line culture medium. Cell cycle analysis of A375 cells exposed to 1 μM CX-5461 for 48 h. A375 cells challenged with CX-5461 showed a significant increase in G2M blockade compared to untreated control.

CaSki vs CX-5461 24hr, 28hr & 72hr Western blots

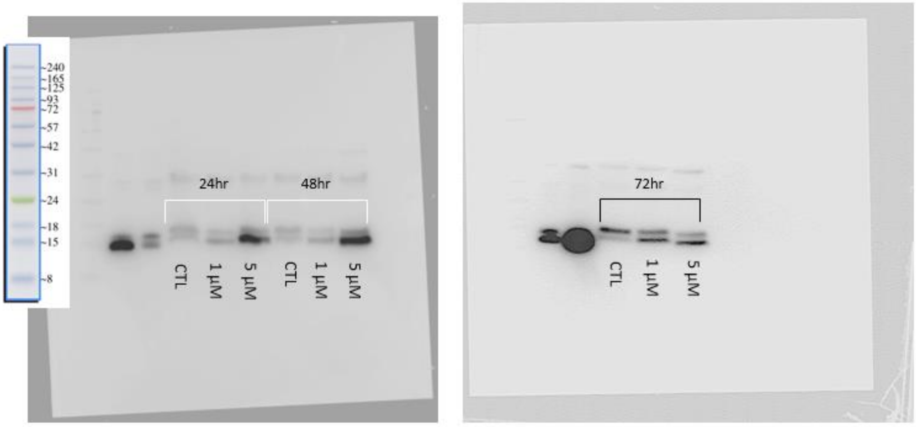

pH2AX CaSKi 48hr and 72hr CX-5461 western

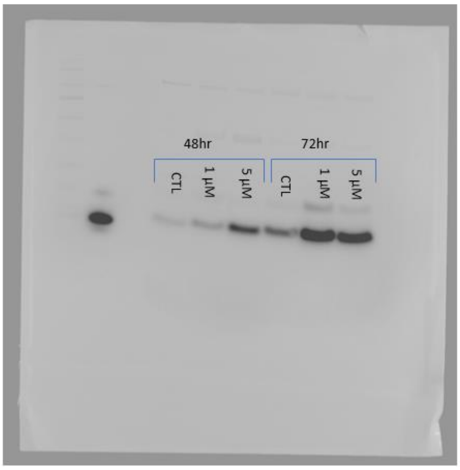

pH2AX CaSKi 48hr and 72hr CX-5461 densitometry

	48hr			72hr		
	CTL	1μM	5μM	CTL	1μM	5μM
Density	3.7009	10.1231	21.6432	3.9803	41.9519	37.0883
Ratio	1	2.7353	5.8481	1	10.5397	9.3178

pH2AX CaSki CX-5461 & X-rays 72hr western

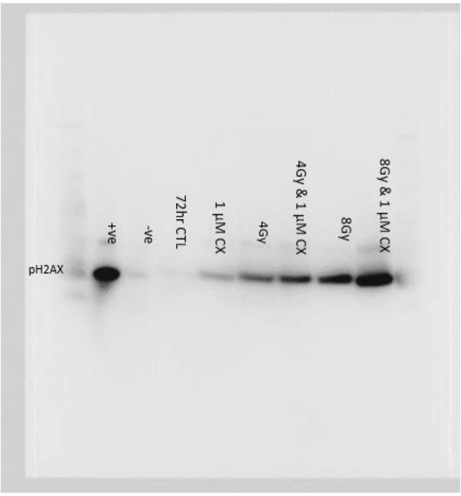

CaSki CX-5461 & X-rays 72hr Densitometry

72hr					
CTL	1µM	4Gy	4Gy 1µM	8Gy	8Gy 1µM
8.9012	23.8431	39.0811	48.6552	47.122	63.1885
1	2.678639	4.390543	5.4661394	5.293893	7.0988743

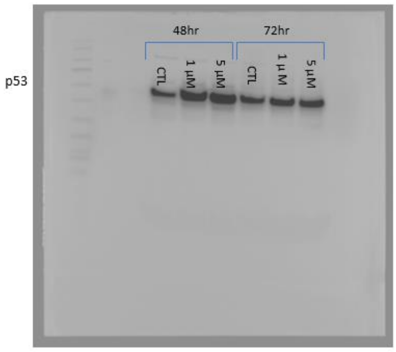

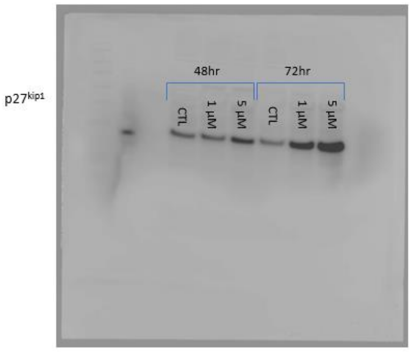

	CASKI p53					
	48hr			72hr		
	CTL	1µM	5µM	CTL	1µM	5µM
Density	48.6468	61.1155	63.5744	47.3641	48.1862	53.2072
Ratio	1	1.256311	1.306857	1	1.017357	1.123366

	CASKI p27					
	48hr			72hr		
	CTL	1µM	5µM	CTL	1µM	5µM
Density	35.8762	37.5821	46.7524	31.8513	52.7548	63.206
Ratio	1	1.04755	1.303159	1	1.656284	1.984409

Figure S3. Detailed information of western blot analysis.

Table S1. CaSki LC3 western band intensity ratios.

**24 h**	**Control**	**1 µM**	**5 µM**
LC3 I Density	31.7675	23.9522	52.1721
LC3 II Density	25.9039	38.8133	85.4466
LC3 II:I Ratio	1	0.753984	1.642311
	0.615421	1.221793	2.689749
II:I Intensity ratio	1	2.633074767	2.661239993
**48 h**	**Control**	**1 µM**	**5 µM**
LC3 I Density	20.7186	17.583	51.1265
LC3 II Density	23.0428	27.3585	92.6294
LC3 II:I Ratio	1	0.848658	2.467662
	0.500000	1.555962473	1.811768792
II:I Intensity ratio	1	3.111924946	3.623539584

72 h	Control	1 μM	5 μM
LC3 I Density	68.4752	63.8067	51.2878
LC3 II Density	55.8014	75.6569	68.9298
LC3 II:I Ratio	1	0.931822	0.748998
	0.514914	1.509766036	1.343980892
II:I Intensity ratio	1	2.932074164	2.610107498

Table S2. List of solid cancer cell lines used for development of panel exposed to CX-5461.

Cell Line Culture Medium & Tissue Type Supplements	
CaSki	RPMI 1640 10% FBS-Epidermoid carcinoma of the cervix
**A375**	DMEM 10% FBS-Malignant melanoma of the skin
**LN18**	DMEM 5% FBS-Glioblastoma of the brain – right temporal lobe
**SKMEL-28**	RPMI 1640 10% FBS-Malignant melanoma of the skin
**HCT-116**	McCoy's A 10% FBS-Colorectal carcinoma of the colon
**G361**	McCoy's A 10% FBS-Malignant melanoma of the skin
**Panc-1**	DMEM 10% FBS-Epithelial carcinoma of the pancreas
**SCaBR**	EMEM 10% FBS-Squamous cell carcinoma of the bladder
**A549**	F-12K 10% FBS-Epithelial carcinoma of the lung
**BXPC3**	RPMI 1640 10% FBS-Adenocarcinoma of the pancreas
**PSN-1**	RPMI 1640 10% FBS-Adenocarcinoma of the pancreas
**PEO1**	RPMI 1640 10% FBS-Adenocarcinoma of the ovary
**PEO1 CarbR**	RPMI 1640 10% FBS-Adenocarcinoma of the ovary
**SiHa**	EMEM 10% FBS-Squamous cell carcinoma of the cervix

© 2019 by the authors. Submitted for possible open access publication under the terms and conditions of the Creative Commons Attribution (CC BY) license (http://creativecommons.org/licenses/by/4.0/).
